# When AI Becomes the First Consultant: Implications for Medical Training

**DOI:** 10.3390/healthcare14213606

**Published:** 2026-10-24

**Authors:** Esteban Ortiz-Prado, Jorge Vasconez-Gonzalez

**Affiliations:** One Health Research Group, Universidad de Las Américas, Quito 170517, Ecuador

**Keywords:** artificial intelligence, clinical reasoning, medical education, critical thinking, medical training

## Abstract

Artificial intelligence enables rapid and easy access to scientific information, allowing for quick diagnostic outputs. As a result, it is becoming a primary source of consultation for medical students. This shift is transforming how clinical knowledge is applied and raises concerns about the development of clinical reasoning. Excessive reliance on these tools may reduce students’ active engagement, limit their diagnostic creativity, and promote a more passive learning style. Furthermore, AI performance can be limited in complex or uncertain clinical scenarios. Therefore, AI should be used as a complementary educational tool that supports the development of autonomous clinical skills, rather than replacing clinical reasoning. It is also essential to teach medical students the responsible use of these technologies, as well as the potential consequences of their misuse.

## 1. Introduction

Artificial Intelligence (AI) refers to the ability of algorithms integrated into systems and tools to learn from data and thus perform automated tasks. Generative AI is a category of AI techniques in which algorithms are trained on datasets that can be used to generate new content, such as text, images, or video [1]. Clinical decision support systems (CDSS), in turn, aim to improve healthcare delivery by optimizing medical decisions using specific clinical knowledge, patient information, and other health information [2]. Meanwhile, AI-powered simulations leverage machine learning algorithms, natural language processing, virtual reality, and adaptive learning systems to create dynamic, personalized, and intelligent learning environments [3].

AI tools are rapidly becoming the first point of consultation for many medical students, due to the speed and ease of access to scientific information, as well as the ability of these tools to provide personalized explanations [4]. AI as the first point of consultation refers to the use of a generative AI system as the first information source consulted when a student encounters an academic, biomedical, or clinical question, before consulting conventional information sources such as faculty members, peers, textbooks, scientific databases, clinical guidelines, or general web-search resources. Faced with a clinical scenario, students can now input symptoms into an AI system and obtain a differential diagnosis within seconds—often more structured and, in some cases, more accurate than their own initial reasoning. While this represents a powerful educational aid, it raises a fundamental question: what kind of cognitive skills are we reinforcing in future physicians? The use of AI and its effects on clinical reasoning must also be distinguished according to the stage at which it is used, whether for formulating differential diagnoses, analyzing and suggesting therapeutic recommendations, or establishing the final diagnosis. Depending on the stage of use, it may limit the student’s active participation in analyzing clinical information, such as signs, symptoms, or medical history, thereby limiting opportunities to generate hypotheses, comparing them with the available information, and subsequently accept them.

**Figure 1:**
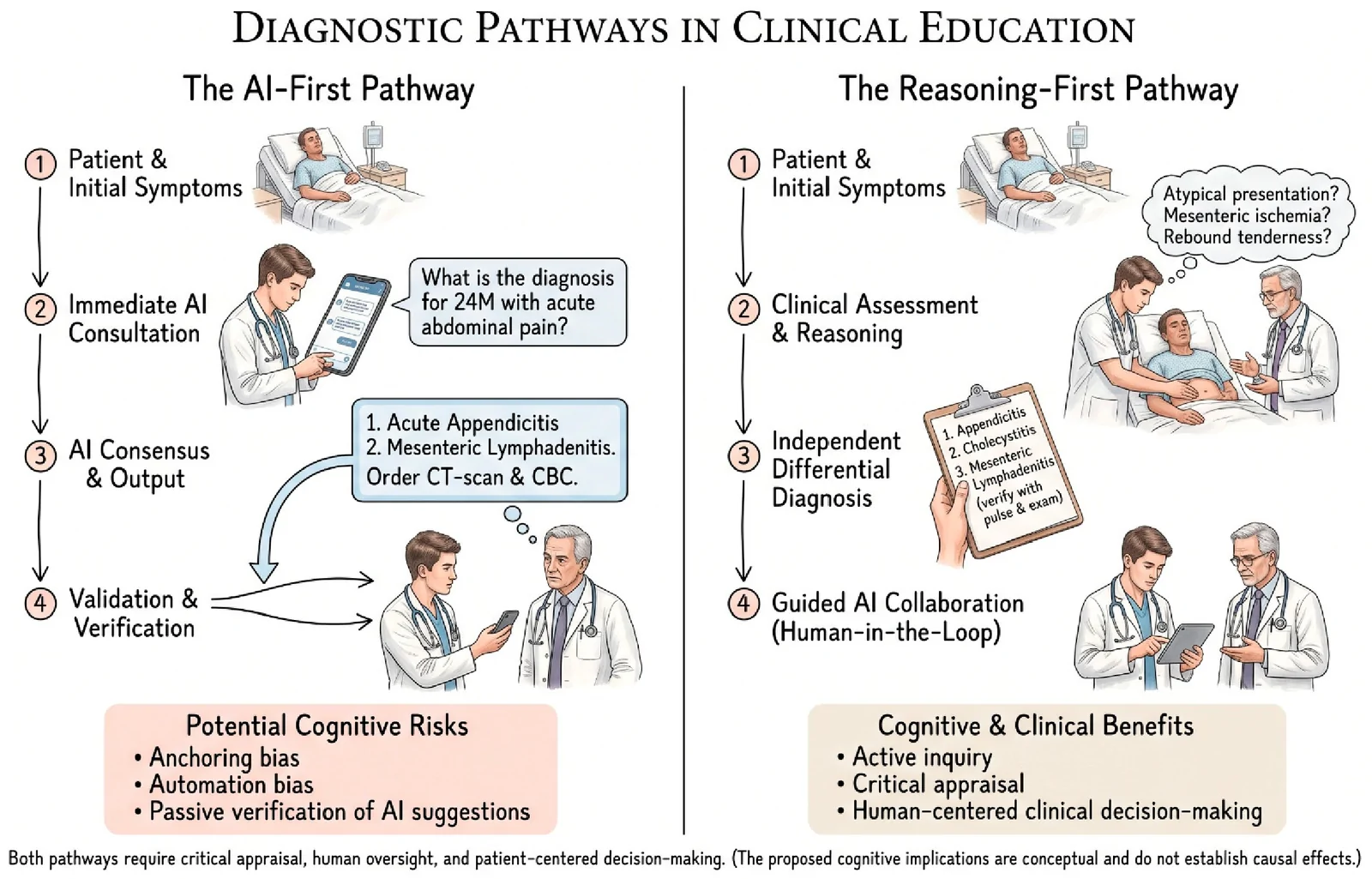
Conceptual comparison of AI-first and reasoning-first pathways in clinical education. In the AI-first pathway, learners consult artificial intelligence before independently developing diagnostic hypotheses, potentially increasing susceptibility to anchoring, automation bias, and passive verification of AI-generated suggestions. In the reasoning-first pathway, learners initially interpret clinical information, formulate differential diagnoses, and evaluate supporting evidence before using AI to compare, challenge, and refine their reasoning. Both pathways require critical appraisal, human oversight, and patient-centered decision-making. The proposed cognitive implications are conceptual and should not be interpreted as established causal effects. The figure was developed with the assistance of generative AI and reviewed by the authors for scientific accuracy (The proposed approaches are conceptual rather than experimentally validated models of cognitive performance).

Clinical training has traditionally been based on the development of reasoning through uncertainty and has relied on classroom learning, cadaver dissections, clinical practice, and hands-on experience with patients [5]. These approaches aim to help medical students develop critical thinking, which is considered one of the most important skills required to provide high-quality medical care [6]. Students are expected to formulate hypotheses, assess probabilities, and refine their reasoning through questioning, feedback, and experience. This process is not limited to reaching the correct diagnosis, but also involves understanding how that diagnosis is constructed. However, because AI has the capacity to replicate aspects of human intelligence, such as learning, reasoning, and problem-solving [7], it may become the first step in clinical decision-making rather than a complementary tool. As a result, opportunities for students to actively engage in the reasoning process may be reduced, potentially leading to excessive dependence on these technologies [5,8]. It is advisable to implement a reasoning-first approach through the use of clinical case studies. Students should identify and analyze relevant signs and symptoms, establish differential diagnoses, and justify their clinical reasoning. Subsequently, students would be allowed to consult an AI tool to receive feedback, explore alternative diagnoses, and obtain recommendations. Finally, they would compare the results, identifying areas of agreement and discrepancy (Figure 1).

A study conducted in Nigeria revealed that 44% of the students surveyed reported the use of AI during their clinical training [9], while in Malaysia, it was observed that 80.6% of health sciences students had frequent practices related to AI, and 96.8% had a positive attitude toward its use [10]. In Pakistan, 68.7% supported its role in the selection of healthcare interventions [11]. On the other hand, Robleto et al. revealed that 96% of medical students reported that Glass AI increased their confidence in diagnosis [12]. Shi et al. observed that most of the medical students surveyed reported regularly using AI tools for academic work and daily tasks, with an average usage frequency of 5.06 times per week. Additionally, 90% reported using two or more AI platforms [13]. A study conducted in Austria involving 438 students found that 80% had used artificial intelligence for their studies or assignments, and 95% supported its use in medical education. The most common applications were explaining concepts and answering questions about medical content [14]. These findings reveal a strong interest in the use of AI among medical students. Each study evaluates different dimensions, such as frequency of use, attitudes, and perceived benefits, among others. However, these measures and study results should not be interpreted as direct evidence of cognitive dependence or impaired clinical reasoning. The early integration of AI into medical education raises questions about its influence on the development of diagnostic reasoning. In a randomized trial involving 111 first- and second-year medical students, misleading AI explanations were found to significantly degrade diagnostic accuracy and showed no evidence of confidence calibration, such that confidence did not reliably distinguish correct from incorrect answers [15]. Similarly, Hayden et al. point out that students do not appear to reliably distinguish between the beneficial and harmful influence of AI, which is consistent with automation bias [16]. For its part, anchoring bias significantly influences clinical decisions, contributes to up to 75% of diagnostic errors, and consists of physicians focusing on a single piece of information, often the initial one, when formulating a diagnosis, without sufficiently adjusting it in light of subsequent information [17,18].

## 2. Impact of AI on Clinical Reasoning and Critical Thinking

Cognitive Load Theory describes how working memory processes information and comprises three types of cognitive load: intrinsic, extraneous, and germane [19]. AI has the potential to reduce unnecessary cognitive load, facilitating the rapid review and assimilation of critical information required to perform tasks essential for safe patient care [20]. Ejaz et al., in their study, report that the frequent use of AI tools is associated with significantly lower cognitive load during complex tasks (β = −0.37, *p* < 0.01), but also with slightly lower self-assessed critical thinking skills (β = −0.21, *p* < 0.05). The authors therefore suggest that, although AI assistance may reduce mental effort, it may also inadvertently discourage deeper engagement with information [21].

However, reliance on AI may lead to several challenges. One immediate consequence is the potential decline in creativity in clinical thinking, which could hinder the development of essential practical clinical skills, diagnostic reasoning, and human-centered decision-making [22]. Instead of exploring multiple diagnostic pathways or considering atypical presentations, students may default to AI-generated outputs. This reliance can further compromise the development of essential clinical competencies, including practical skills, diagnostic reasoning, and patient-centered decision-making [23]. On the other hand, a dependency on AI for problem-solving and learning processes may develop, which could reduce participation in critical thinking and independent learning. Furthermore, such dependency may also include tasks such as taking notes or receiving instant diagnostic suggestions, which can reduce active participation and patient engagement during clinical encounters [24]. It has been observed that misleading explanations provided by AI reduced diagnostic accuracy and showed no evidence of students’ confidence calibration, meaning that confidence did not reliably distinguish correct answers from incorrect ones [15]. A study analyzing the impact of AI-generated responses from ChatGPT on medical students’ decision-making found that AI can significantly influence students’ decision-making. In addition, misconceptions or misinformation from natural language processors may be considered more influential than peer discussion [25]. On the other hand, a systematic review evaluating higher-order clinical reasoning and complex decision-making reported conflicting results: one study found no overall differences, whereas others suggested lower performance or reduced engagement when AI-supported approaches were used [26]. Tay et al., in their systematic review and meta-analysis, concluded that AI-based educational interventions may improve critical thinking and clinical reasoning among healthcare students; however, the certainty of the evidence was very low [27]. Similarly, Alhur et al., in their systematic review, reported null, mixed, and negative results, particularly when AI was compared with established educational resources or used without structured instructional guidance [28].

Over time, this may lead to a more passive learning style, where the emphasis shifts from inquiry and interpretation to verification and acceptance. The ability to ask the right clinical questions—a cornerstone of medical practice—risks being replaced by the ability to formulate effective prompts.

Moreover, while AI may improve diagnostic accuracy in controlled or well-represented conditions, its performance in scenarios involving diagnostic uncertainty remains suboptimal compared with that of physicians [29]. This is particularly concerning in situations where AI-generated outputs are biased, incomplete, or not aligned with local epidemiological realities. In a study that evaluated the longitudinal clinical reasoning capabilities of state-of-the-art Large Language Model (LLMs), it was observed that state-of-the-art LLMs achieved high accuracy in final diagnoses but performed poorly in generating differential diagnoses and managing uncertainty [30]. Furthermore, when presented with incorrect data and information, students, lacking the necessary experience to discern and identify such errors, may assume that the information is reliable, thereby affecting diagnostic accuracy.

Another critical issue is the implicit assumption of constant access. The growing integration of AI in medical education suggests a future in which students—and eventually physicians—are always connected and always supported by digital tools. While this may be realistic in high-resource settings, it is far from guaranteed in many parts of the world. In low- and middle-income countries, and especially in rural or underserved regions, access to stable internet, devices, or AI platforms cannot be assumed [31,32]. In such contexts, overreliance on AI during training may leave future physicians underprepared to practice independently. This raises a broader concern about equity. If medical education increasingly incorporates AI-dependent learning strategies, students without consistent access to these tools may be disadvantaged [33]. At the same time, those who do rely heavily on AI may develop skills that are not transferable to settings where such technology is unavailable. In both cases, disparities are reinforced. In low-resource countries, various strategies must be implemented to improve the resilience of digital infrastructure, such as adopting hybrid or solar-powered energy systems, implementing offline-capable AI models with local data storage, and building local technical maintenance capacity through specialized staff training [34]. Furthermore, medical education in these settings can preserve high-quality clinical reasoning training without relying on AI tools through active, student-centered educational strategies, such as case-based learning, illness script teaching, simulation, and deliberate practice with feedback, which have been associated with improvements in clinical reasoning and diagnostic performance [35].

Nevertheless, not all findings are negative, and it is also important to highlight the positive aspects of AI use in medical education. To this end, it is first necessary to understand that diagnostic reasoning consists of two distinct components: an early phase in which the clinician formulates one or more diagnostic hypotheses, and a subsequent phase in which these hypotheses are systematically tested [36]. According to the Dual Process Theory of Clinical Reasoning, clinical reasoning begins with System 1 processes, in which the physician evaluates the symptoms presented by the patient, as well as other clinical evidence, to arrive at a differential diagnosis [37]. If necessary, additional clinical evidence is acquired and analyzed through System 2 processes to evaluate the differential diagnosis, until a clinical decision is made that diagnoses the patient’s disease and determines the best therapeutic course of action [37]. In a study in which one group of primary care professionals received training through a traditional face-to-face training activity and another group received online training through feedback provided by an artificial intelligence-powered simulation, no significant differences in diagnostic performance were observed before their respective interventions. After the intervention, the diagnostic performance of the second group improved by 22% (*p* < 0.02; Cohen’s d, effect size = 0.79) [38]. On the other hand, excessive dependence on AI occurs when users accept AI-generated recommendations without questioning them, which in turn leads to errors in task performance in the context of decision-making [39]. This is because they have difficulty assessing the reliability of AI or the degree of trust they should place in its suggestions [39]. AI affects cognitive abilities, as users increasingly prefer quick and optimal solutions over slower ones, limited by practicality [39].

Nevertheless AI can serve as an interactive support tool for medical students by acting as a personalized tutor outside the classroom, automatically generating flashcards, simplifying complex concepts, and providing immediate feedback [40]. Additionally, clinical simulators can create a stress-free environment in which medical students can practice clinical reasoning and learn from their mistakes. AI also have the ability to adapt educational content and assessments to individual learners’ needs, thereby enabling more efficient knowledge acquisition and deeper conceptual understanding [22]. AI has been reported to reduce reliance on incomplete heuristics and potentially mitigate cognitive biases, while providing consistent performance unaffected by fatigue or information overload [41]. It is important to note that the findings of the cited articles should not be interpreted as direct evidence that AI use causes a decline in creativity, learning, or clinical reasoning skills among medical students. Therefore, further research is needed to determine whether sustained, long-term AI use affects the development of these skills in medical students.

## 3. Strategies for Responsible Integration of AI in Medical Education

Importantly, this is not an argument against the use of AI in medical education. On the contrary, AI has the potential to enhance learning, democratize access to information, and support clinical training. This is demonstrated by Dahò and Caci in their study, which examined whether generative AI could serve as a meaningful complementary tool for clinicians in designing individualized cognitive rehabilitation plans. The authors concluded that AI-assisted rehabilitation design appears to be a promising field, highlighting the importance of structured prompt design and systematic expert evaluation to ensure the clinical soundness of AI-generated materials. Furthermore, prompts should be formulated based on established therapeutic reasoning, and expert review remains essential for assessing both the quality and applicability of AI-generated outputs [42]. AI should complement—not replace—the cognitive processes that underpin clinical reasoning. Educational strategies must therefore evolve to ensure that students continue to develop independent analytical skills. This may include structured use of AI after initial hypothesis generation, critical appraisal of AI outputs, and explicit training on the limitations and biases of these systems. The concept of “human-in-the-loop” is essential, particularly in educational contexts where the goal is not only to obtain correct answers but to train competent, adaptable clinicians.

Other recommendations include developing AI literacy so that students can critically evaluate AI outputs, as well as recognize and be aware of the limitations of these tools [24]. AI-assisted case generation, in which interactive virtual patients are created, encourages students to justify diagnostic hypotheses, interpret investigations, or refine management plans, as well as collect clinical histories and formulate differential diagnoses [43]. Furthermore, to assess reductions in the quality of reasoning or creativity over time among those who prioritize the use of AI, the Revised-IDEA (R-IDEA) score can be used, a validated 10-point scale evaluating 4 core domains of clinical reasoning documentation [44]. The Script Concordance Test (SCT) can also be used, which evaluates clinical reasoning in a context of uncertainty by comparing examinees’ responses with those of expert physicians [45]. Additionally, it would be advisable to establish practical assessments to analyze and compare students’ clinical reasoning before and after using any AI tool. For this purpose, clinical cases can be established. In the first round, students should analyze the case, establish differential diagnoses, and justify their initial diagnosis. In the second round, they would then use AI to obtain recommendations and make any modifications. Once both phases have been completed, a comparison would be conducted using rubrics to analyze the appropriateness of the established diagnoses and identify potential errors. Other tools, such as oral examinations, can also be implemented to assess independent reasoning, since students cannot use any type of AI during these assessments.

Additionally, it is important to note that when implementing AI-supported medical reasoning training, differences in access to the Internet, electricity, and digital devices must be taken into account. Therefore, in contexts with unstable or intermittent Internet connectivity, AI could be used asynchronously as a complementary tool to provide feedback or support.

On the other hand, institutions implementing AI-supported medical reasoning training programs should develop protocols for interaction with AI, explicitly defining permitted and recommended behaviors [46]. It is also necessary to establish guidelines regarding data use, including what types of information can and cannot be shared with AI systems [46]. In addition, AI tools should be monitored and evaluated frequently [47]. Furthermore, AI literacy must be developed to prepare students for ethical and effective interaction with emerging technologies [48]. It is also necessary to actively promote equity by identifying and correcting discriminatory outputs in AI-generated content [49].

It is important to note that clinical reasoning is an iterative process involving hypothesis generation and data collection. Furthermore, diagnostic performance depends on domain knowledge, pattern recognition, contextual cues, and the ability to recognize when initial impressions require revision [50]. Therefore, the implementation of AI as a complementary pedagogical tool in activities aimed at developing clinical reasoning requires strategies that promote active cognitive engagement, critical reflection, and the independent formulation of hypotheses by medical students. Students must also be able to evaluate AI-generated information and contrast it with scientific evidence, rather than simply assuming that all AI-generated information is accurate. Furthermore, supervision of its use is necessary to maintain human responsibility for clinical judgment.

## 4. Conclusions

The widespread adoption of AI by medical students is transforming the way clinical knowledge is accessed and applied in practice. Although it offers undeniable advantages, it also poses risks to the development of creativity, reasoning, and autonomy. As we integrate AI into medical education, we must ask not only what students are learning, but also how they are learning and what skills they might be losing in the process. However, the aim is not to discourage the use of artificial intelligence, but rather to promote its responsible use by establishing guidelines under which these technologies can be used to their fullest potential in support of learning, without compromising or affecting the development of students’ clinical judgment. Likewise, equitable implementation and access are important, particularly in resource-limited settings. For this reason, educational institutions must adopt approaches tailored to different contexts for the integration of AI while preserving traditional educational practices.

## Data Availability

No new data were created or analyzed in this study. Data sharing is not applicable to this article.

## Abbreviations

The following abbreviations are used in this manuscript:

AI	Artificial intelligence
LLMs	Large Language Model
R-IDEA	Revised-IDEA
SCT	Script Concordance Test
